# Long noncoding RNA LINC01291 promotes the aggressive properties of melanoma by functioning as a competing endogenous RNA for microRNA-625-5p and subsequently increasing IGF-1R expression

**DOI:** 10.1038/s41417-021-00313-9

**Published:** 2021-03-05

**Authors:** Lijun Wu, Ke Li, Wei Lin, Jianjiang Liu, Qiang Qi, Guoliang Shen, Weixin Chen, Wenjun He

**Affiliations:** 1 Department of Plastic and Aesthetic Surgery, The Second Affiliated Hospital of Soochow University, Jiangsu, China; 2 Department of Burn and Plastic Surgery, The First Affiliated Hospital of Soochow University, Jiangsu, China

**Keywords:** Melanoma, Cell biology

## Abstract

Studies have confirmed the relationship between dysregulated long noncoding RNAs and melanoma pathogenesis. However, the regulatory functions of long intergenic non-protein coding RNA 1291 (LINC01291) in melanoma remain unknown. Therefore, we evaluated LINC01291 expression in melanoma and explored its roles in regulating tumor behaviors. Further, the molecular events via which LINC01291 affects melanoma cells were investigated. LINC01291 expression in melanoma cells was analyzed using The Cancer Genome Atlas database and quantitative real-time polymerase chain reaction. Functional assays, including the Cell Counting Kit-8 assay, colony formation assay, flow cytometry, cell migration and invasion assays, and tumor xenograft models, were used to examine LINC01291’s role in melanoma cells. Additionally, bioinformatics analysis, RNA immunoprecipitation, luciferase reporter assay, and western blotting were conducted to determine the tumor-promoting mechanism of LINC01291. LINC01291 was upregulated in melanoma tissues and cell lines. Following LINC01291 knockdown, cell proliferation, colony formation, migration, and invasion were diminished, whereas apoptosis was enhanced and the cell cycle was arrested at G0/G1. In addition, loss of LINC01291 decreased the chemoresistance of melanoma cells to cisplatin. Furthermore, LINC01291 interference inhibited melanoma tumor growth in vivo. Mechanistically, LINC01291 functions as a competing endogenous RNA by sponging microRNA-625-5p (miR-625-5p) in melanoma cells and maintaining insulin-like growth factor 1 receptor (IGF-1R) expression. Rescue experiments revealed that the roles induced by LINC01291 depletion in melanoma cells could be reversed by suppressing miR-625-5p or overexpressing IGF-1R. Our study identified the LINC01291/miR-625-5p/IGF-1R competing endogenous RNA pathway in melanoma cells, which may represent a novel diagnostic biomarker and an effective therapeutic target for melanoma.

## Introduction

Melanoma originates from the malignant transformation of melanocytes located at the basement of the epidermis. It is the most common and malignant form of human skin cancer [1, 2]. Although it accounts for only 4% of skin cancer cases, is responsible for approximately three-quarter of the mortalities resulting from skin cancer [3]. Over the past several decades, the incidence and mortality of melanoma have seen a significant upward trend [4]. At present, complex treatment, including surgical resection, biotherapy, radiochemotherapy, targeted therapy, and immunotherapy, are widely used to treat melanoma. These treatment modalities have significantly improved the curative effect and prolonged survival time [5]. Nevertheless, significant improvements are still needed in terms of the clinical outcomes of patients with the advanced-stage disease [6]. Melanoma has a tendency to metastasize, and patients with distant metastasis exhibit poor prognosis [7]. Therefore, it is of paramount importance to identify the underlying molecular mechanisms of melanoma pathogenesis to develop effective strategies for treatment.

Long noncoding RNAs (lncRNAs) represent a family of RNA transcripts that are over 200 nucleotides in length [8]. They are considered to be untranslated RNA molecules; therefore, they do not encode proteins [9]; however, they have the ability to interact with DNA, RNA, or proteins to regulate gene expression at the transcriptional or post-transcriptional level [10]. Increasing evidence suggests a relationship between lncRNAs and a variety of cellular processes [11–13]. Notably, several studies have indicated that lncRNAs exert cancer-suppressing or cancer-promoting activities and perform diverse functions in the oncogenesis and progression of human cancers [14–16]. With respect to melanoma, several lncRNAs are abnormally expressed and may exert regulatory effects on tumor-related processes [17–19].

MicroRNAs (miRNAs), a group of noncoding RNA molecules that are 17–24 nucleotides in length, negatively regulate gene expression by complementary base-pairing with the 3′-untranslated regions (3′-UTRs) of their target genes; this results in mRNA degradation and/or translational suppression [20]. As a result, miRNAs are efficient regulators of cancer progression and have been implicated in a wide range of aggressive tumor behaviors [21]. A recent study provided a novel regulatory mechanism theory involving lncRNAs and miRNAs and their targeted genes [22]. They proposed that lncRNAs function as competing endogenous RNAs (ceRNAs) to sequester miRNAs that share common miRNA response elements. This suppresses the regulatory effect of miRNAs on their targeted genes and ultimately attenuates the etiology and progression of cancers [23–25]. Therefore, deciphering the ceRNA pathway in melanoma may provide novel targets for melanoma diagnosis and lead to the development of new therapies.

While lncRNAs are well-known for their pivotal role in human malignancies, the regulatory functions of the long intergenic non-protein coding RNA 1291 (LINC01291) in melanoma have not yet been fully investigated. Therefore, the present study attempted to comprehensively explore the roles and related mechanisms of LINC01291 in melanoma.

## Subjects and methods

### Patients and clinical specimens

This study was approved by the Ethics Committee of the First Affiliated Hospital of Soochow University and was performed according to the tenets of the Declaration of Helsinki. All participants read and signed informed consent agreements before their inclusion in the study. Human melanoma tissues and corresponding adjacent normal tissues were collected from 41 patients with melanoma at the First Affiliated Hospital of Soochow University. The diagnosis of melanoma was confirmed pathologically. The histological characteristics of tissue were independently diagnosed by two pathologists. Exclusion criteria were as follows: patients with other types of cancer and those who had previously received radiochemotherapy, radiotherapy, immunotherapy, or any other anticancer therapies. Tissues were excised and stored in liquid nitrogen until further analysis.

### Cell lines

The A-375 human melanoma cell line was obtained from the Shanghai Institute of Cell Biology at the Chinese Academy of Sciences (Shanghai, China) and cultured in Dulbecco’s modified Eagle’s medium (DMEM; Gibco; ThermoFisher Scientific, Inc., Waltham, MA, USA) supplemented with 10% fetal bovine serum (FBS; Gibco; ThermoFisher Scientific), 1% sodium pyruvate (Gibco; ThermoFisher Scientific), and 1% penicillin/streptomycin (Gibco; ThermoFisher Scientific). A-375 cell line is a suitable transfection host. This cell line is also the ideal control for NRAS mutant-A-375 isogenic cell line

Three additional melanoma cell lines, namely HT-144, SK-MEL-1, and A2058, were purchased from the American Type Culture Collection (Manassas, VA, USA). These three cell lines were maintained in McCoy’s 5a medium (Gibco; ThermoFisher Scientific), minimum essential medium (Gibco; ThermoFisher Scientific), and DMEM, respectively, all of which were supplemented with 10% FBS and 1% penicillin/streptomycin. HT-144 was derived from metastatic site and subcutaneous tissues, while SK-MEL-1 was derived from metastatic site and lymphatic system. A2058, derived from a lymph node, is a BRAF genetic alteration cell panel.

Human epidermal melanocytes (HEMs) were purchased from ScienCell Research Laboratories, Inc. (San Diego, CA, USA) and maintained in melanocyte medium (ScienCell Research Laboratories, Inc.). All cells were identified by short tandem repeat genotyping and tested for mycoplasma contamination. All cells were grown in a humidified incubator at 37 °C with 5% CO_2_.

### Oligonucleotides, plasmids, and cell transfection

Specific small interfering RNAs (siRNAs) targeting LINC01291 (si-LINC01291) and non-targeted siRNA (si-NC) were designed and synthesized by GenePharma (Shanghai, China). The si-LINC01291#1 sequence was 5′-CTGCATCAATTTGATAAATAATC-3′; si-LINC01291#2 sequence was 5′-TGCATCAATTTGATAAATAATCC-3′; si-LINC012911#3 sequence was 5′-ACCTTTCAAACGGAAATAAAACT-3′; and the si-NC sequence was 5′-CACGATAAGACAATGTATTT-3′. miR-625-5p mimic, negative control (NC) mimic, miR-625-5p inhibitor (anti-miR-625-5p), and NC inhibitor (anti-NC) were obtained from RiboBio (Guangzhou, China). The insulin-like growth factor 1 receptor (IGF-1R) overexpression plasmid pcDNA3.1-IGF-1R (pc-IGF-1R) was constructed by Shanghai Sangon Biotech Co., Ltd (Shanghai, China). Empty pcDNA3.1 plasmid was used as a control. Cells were seeded into 6-well plates. The next day, the oligonucleotides or plasmids were transfected into cells using Lipofectamine 2000 reagent (Invitrogen; ThermoFisher Scientific, Inc.) according to the manufacturer’s instructions.

### RNA isolation and quantitative real-time polymerase chain reaction (qRT-PCR)

Total RNA was extracted using the TRIzol reagent (Invitrogen; ThermoFisher Scientific, Inc.) and quantified using the NanoDrop 1000 spectrophotometer (NanoDrop Technologies; ThermoFisher Scientific, Inc.). To determine miR-625-5p and miR-766-5p expression, the miRcute miRNA First-Strand cDNA Synthesis Kit (Tiangen Biotech, Beijing, China) was used to synthesize first-strand cDNA from total RNA. Quantitative PCR was performed using the miRcute miRNA qPCR Detection Kit SYBR Green (Tiangen Biotech). Relative miR-625-5p and miR-766-5p expression were normalized to that of U6 small nuclear RNA. To quantify the expression of LINC01291 and IGF-1R, reverse transcription was performed using the PrimeScript™ RT reagent kit with gDNA Eraser (Takara Biotechnology Co., Ltd., Dalian, China), followed by quantitative PCR using the TB Green® Premix Ex Taq™ II (Takara Biotechnology Co., Ltd.). GAPDH served as the control for the expression of LINC01291 and IGF-1R. Gene expression was calculated by the 2^−ΔΔCt^ method.

The primers were designed as follows: LINC01291, 5′-ATTCATCACCTTTCAAACGGAAA-3′ (forward) and 5′-CATGGTGAACTTGGTTCAAATGT-3′ (reverse); IGF-1R, 5′-ATCTCAAGGATATTGGGCTTTACAA-3′ (forward) and 5′-CCAGTCCACAGTGGAGAGGTAAC-3′ (reverse); GAPDH, 5′-CGGAGTCAACGGATTTGGTCGTAT-3′ (forward) and 5′-AGCCTTCTCCATGGTGGTGAAGAC-3′ (reverse); miR-625-5p, 5′- TCGGCAGGAGGGGGAAAGU-3′ (forward) and 5′- CACTCAACTGGTGTCGTGGA-3′(reverse); miR-766-5p, 5′-TCGGCAGGAGGAGGAAUUGG-3′ (forward) and 5′-CACTCAACTGGTGTCGTGGA-3′(reverse); and U6, 5′-GCTTCGGCAGCACATATACTAAAAT-3′ (forward) and 5′-CGCTTCACGAATTTGCGTGTCAT-3′ (reverse).

### Subcellular fractionation assay

Exponentially growing melanoma cells were harvested and subjected to subcellular fractionation using the Cytoplasmic & Nuclear RNA Purification kit (Norgen Biotek Corp.). Then, qRT-PCR was performed to determine the relative distribution of LINC01291 in the nuclear and cytoplasmic fractions. U6 and GAPDH were used as controls for the nuclear and cytoplasmic fractions, respectively.

### Cell Counting Kit-8 (CCK-8) and colony formation assays

Transfected cells were seeded into 96-well plates and grown in an incubator at 37 °C for 3 days. Cellular proliferation was determined every 24 h using the CCK-8 kit (Dojindo Molecular Technologies, Japan). Briefly, 10 μl of the CCK-8 reagent was added to each well, followed by incubation of the plates for 2 h at 37 °C. Cell proliferation was assessed by measuring the absorbance at 450 nm using a microplate spectrophotometer.

For the colony formation assays, transfected cells were harvested at 24 h post-transfection and resuspended into a fresh culture medium. The cell suspension (2 ml) containing 1000 cells was seeded into 6-well plates and grown in a humidified atmosphere at 37 °C with 5% CO_2_. After 2 weeks, the resulting colonies were fixed with 4% paraformaldehyde and stained with 0.1% crystal violet. The colonies were imaged and counted under an inverted light microscope (Olympus Corporation, Tokyo, Japan).

### CCK-8 assay for drug resistance

Transfected cells were harvested at 24 h pot-transfection and seeded into 96-well plates. Each well covered 100 μl cell suspension containing 2 × 10^3^ cells. After overnight incubation, the adhered cells were cultivated with a series of medium with an increasing dose of cisplatin. Two days later, 10 μl of the CCK-8 reagent was applied in incubating cells for an additional 2 h. The absorbance at 450 nm was detected using a microplate spectrophotometer. The IC50 of cisplatin was then determined.

### Analyses of cell cycle and apoptosis by flow cytometry

The Annexin V-FITC Apoptosis Detection Kit (Beyotime Biotechnology Ltd., Shanghai, China) was used to determine cell apoptosis. After a 24-h incubation period, the cells were washed with phosphate buffer solution (PBS), digested with trypsin, and centrifuged at 1000 × *g* for 5 min. Prior to resuspension into 195 μl of annexin V-FITC binding buffer, the transfected cells were rinsed with PBS, centrifuged, and the supernatant was discarded. The cell suspension was supplemented with 5 µl of annexin V-FITC and 10 µl of propidium iodide (PI). Then, the cells were placed in the dark at 20–25 °C for 15 min. The early and late apoptotic cells were detected via flow cytometry (BD Biosciences, Franklin Lakes, NJ, USA).

To determine the cell cycle status, the transfected cells were collected using the experimental steps described above and fixed in 1 ml of 70% ice-cold ethanol at 4 °C for 2 h. After washing the cells with ice-cold PBS, they were centrifuged at 1000 × *g* for 5 min and resuspended in 425 µl of cell staining buffer, which was supplemented with 50 µl of RNase and 25 µl of PI (all from BioLegend, San Diego, CA, USA). The cell cycle status was analyzed via flow cytometry.

### Cell migration and invasion assays

For the migration assay, the transfected cells were harvested and resuspended in a serum-free culture medium. The cell suspension (200 μl) containing 5 × 10^4^ cells was seeded into the upper Transwell chambers (8 μm pore size; BD Biosciences), whereas the lower chambers contained 500 μl of complete medium. After incubating the cells at 37 °C for 1 day, the non-migrated cells were removed using a cotton swab and the migrated cells were fixed with 4% paraformaldehyde and stained with 0.1% crystal violet. The stained cells were counted under an inverted light microscope at ×200 magnification. Five randomly selected fields were analyzed. For the cell invasion assay, the chambers were pre-coated with Matrigel (BD Biosciences), and the remaining experimental steps were similar to the migration assay.

### Tumor xenograft model

All animal experiments were performed according to the NIH guidelines for the care and use of laboratory animals and were approved by the Committee on the Use and Care of Animals of the First Affiliated Hospital of Soochow University. The short hairpin RNA (shRNA) targeting LINC01291 (sh-LINC01291) and non-targeted shRNA (sh-NC) were designed and synthesized by GenePharma. The sh- LINC01291 sequence was 5′-CCGGCTGCATCAATTTGATAAATAATCCTCGAGGATTATTTATCAAATTGATGCAGTTTTTG-3′; and the sh-NC sequence was 5′-CCGGCACGATAAGACAATGTATTTCTCGAGAAATACATTGTCTTATCGTGTTTTTG-3′. The sh-LINC01291 and sh-NC constructs were inserted into a lentiviral vector and transfected into 293T cells together with the “helper” packaging vectors psPAX2 and pMD2.G. The lentiviruses stably overexpressing either sh-LINC01291 or sh-NC were harvested after 2 days and introduced into A-375 cells. Puromycin (Sigma-Aldrich; Merck KGaA) was used to select stable LINC01291-depleted A-375 cells.

BALB/c nude mice (male; 4 weeks old) were acquired from the Shanghai Experimental Animal Center (Shanghai, China) and kept under specific pathogen-free conditions. All mice were randomized into two groups: sh-LINC01291 and sh-NC groups, with three nude mice in each group. A-375 cells stably expressing sh-LINC01291 or sh-NC were subcutaneously injected into nude mice. Following the injection, the width and length of the resulting tumors were measured every week and tumor volumes were calculated using the formula: volume = 1/2 × length × width^2^. All mice were euthanized via cervical dislocation at 4 weeks after cell injection. The tumor xenografts were dissected and weighed, followed by further IGF-1R, Ki-67, and cleaved caspase-3 immunohistochemical staining.

### Bioinformatics analysis

miRDB (http://mirdb.org/) and ENCORI (http://starbase.sysu.edu.cn/) were used to predict miRNA binding to LINC01291. The potential targets of miR-625-5p were predicted using two online databases, namely miRDB and TargetScan (http://www.targetscan.org/vert_60/).

### RNA immunoprecipitation (RIP) assay

The RIP assay was performed using the EZ-Magna RIP RNA-binding protein immunoprecipitation kit (Millipore, Bedford, MA, USA). Melanoma cells were lysed using RIPA lysis buffer (Beyotime Biotechnology Ltd.). Following centrifugation, the cell extract was collected and a portion of the cell lysate was designated as the input. The remaining cell extract was cultivated in RIP buffer containing magnetic beads that were already conjugated with argonaute-2 (Ago2) antibody or NC IgG (both from Millipore). The magnetic bead–protein complex was collected and incubated with proteinase K to digest the protein. The immunoprecipitated RNA was extracted and subjected to qRT-PCR to detect LINC01291 and miR-625-5p.

### Luciferase reporter assay

LINC01291 fragments containing the presumed wild-type (WT) miR-625-5p binding sequences and mutant (MUT) sites were amplified and inserted into the psiCHECK™-2 luciferase reporter vector (Promega Corporation, Madison, WI, USA) to generate the LINC01291-WT and LINC01291-MUT reporter vectors. The IGF-1R-WT and IGF-1R-MUT reporter vectors were created using the same method. Melanoma cells were seeded into 24-well plates and incubated for 24 h. The WT or MUT reporter vectors were cotransfected with miR-625-5p mimic or NC mimic into melanoma cells using Lipofectamine 2000 reagent. After 48 h, the Dual-Luciferase Reporter Assay System (Promega Corporation) was used to measure luciferase activity.

### Western blot analysis

RIP assay lysis buffer (Beyotime Biotechnology Ltd.) was used to extract total protein. Protein quantification was performed using the Bradford Protein Assay Kit (Beyotime Biotechnology Ltd.,). Equivalent amounts of protein were separated via sodium dodecyl sulfate–polyacrylamide gel electrophoresis. The proteins were transferred onto polyvinylidene difluoride membranes (Beyotime Biotechnology Ltd.) and blocked with 5% skimmed milk. The membranes were then incubated with primary antibodies against IGF-1R (ab182408; 1:1000 dilution; Abcam, Cambridge, MA, USA) or GAPDH (ab181602; 1:1000 dilution; Abcam) overnight at 4 °C. The membranes were incubated with horseradish peroxidase-conjugated anti-mouse IgG secondary antibody (ab205718; 1:5000 dilution; Abcam) followed by detection with the BeyoECL moon protein detection kit (Beyotime Biotechnology Ltd.). GAPDH was used as a loading control.

### Statistical analysis

All experiments were repeated three times and each experiment was performed in triplicate unless otherwise stated. All data are expressed as the mean ± standard deviation and analyzed using the SPSS 24.0 statistical software package (IBM-SPSS Inc., Chicago, IL, USA). All statistical tests were justified as appropriate and the data met the assumptions of the tests. The variance was similar between the groups being statistically compared. Samples and animals were randomly allocated to the experimental groups and processed. Further, they were blinded to the investigator. Sample size estimation tests were not performed for our animal studies. No samples or mice were excluded from the analysis as outliers. The statistical significance between the two groups was calculated using the two-tailed unpaired or paired *t*-tests. One-way analysis of variance followed by Tukey’s post-hoc test was used to determine differences between multiple groups. Gene expression correlation was performed via Pearson’s correlation analysis. *P* < 0.05 was considered statistically significant.

## Results

### LINC01291 depletion inhibits the proliferation, migration, and invasion of melanoma cells but increases cell apoptosis and results in G0/G1 cell cycle arrest

**Fig. 1:**
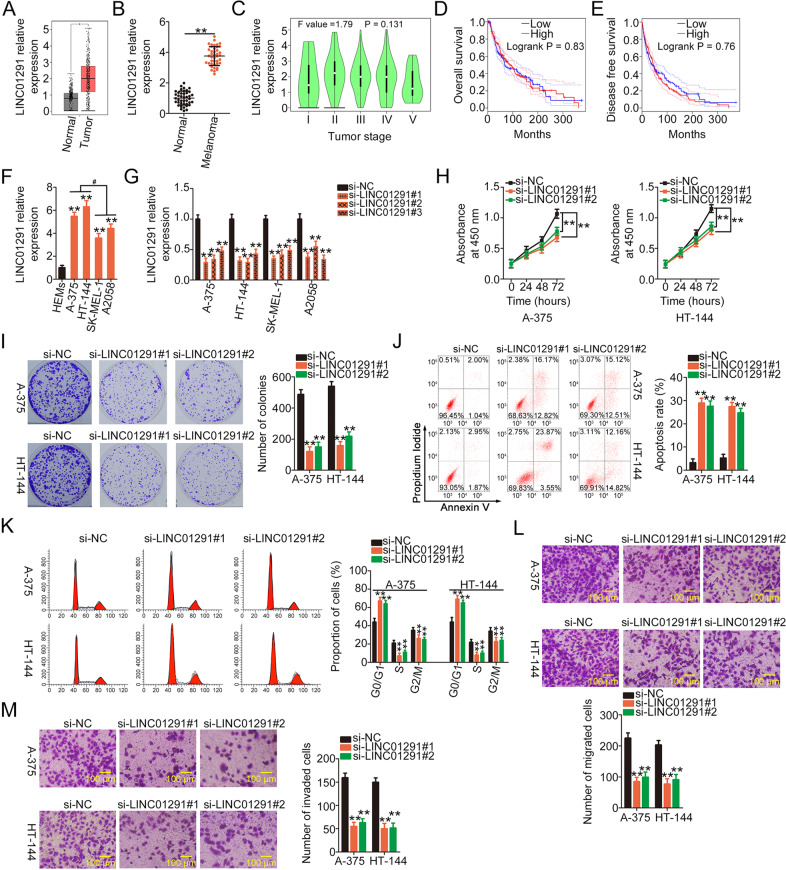
Long intergenic non-protein coding RNA 1291 (LINC01291) depletion results in tumor suppression in melanoma cells. **A** LINC01291 level in melanoma was analyzed using The Cancer Genome Atlas (TCGA) database. **P < 0.01 vs. group “Normal”. **B** LINC01291 expression in 41 pairs of human melanoma tissues and corresponding adjacent normal tissues was measured by quantitative real-time polymerase chain reaction (qRT-PCR). **P < 0.01 vs. group “Normal”. **C**–**E** TCGA database was used to analyze the association between LINC01291 expression and tumor stage, overall survival, and disease-free survival in melanoma cells. **F** qRT-PCR analysis was used to measure LINC01291 expression in four melanoma cell lines (A-375, HT-144, SK-MEL-1, and A2058), with human epidermal melanocytes (HEMs) as a control. **P < 0.01 vs. group “HEMs”. ^#^P < 0.05 vs. cell lines SK-MEL-1 and A2058. **G** A-375 and HT-144 cells were transfected with si-LINC01291 or non-targeted siRNA and the relative expression of LINC01291 was measured by qRT-PCR. **P < 0.01 vs. group “si-NC”. **H**, **I** Cell proliferation and colony formation abilities were determined using the Cell Counting Kit-8 and colony formation assays in A-375 and HT-144 cells after LINC01291 downregulation. **P < 0.01 vs. “si-NC”. **J**, **K** Flow cytometry was used to analyze cell apoptosis and cell cycle status of A-375 and HT-144 cells following transfection with si-LINC01291 or non-targeted siRNA. **P < 0.01 vs. group “si-NC”. **L**, **M** Cell migration and invasion assays revealed the influence of si-LINC01291 transfection on the migration and invasion of A-375 and HT-144 cells. **P < 0.01 vs. group “si-NC”.

First, LINC01291 expression in melanoma was analyzed using The Cancer Genome Atlas (TCGA) database. LINC01291 expression was higher in melanoma tissues (*n* = 466) than in normal (*n* = 558) tissues (Fig. 1A). To verify this observation, LINC01291 expression was measured in 41 pairs of human melanoma tissues and corresponding adjacent normal tissues using qRT-PCR. LINC01291 was overexpressed in melanoma tissues than in adjacent normal tissues (Fig. 1B). The clinical significance of LINC01291 was also evaluated using the TCGA database. However, no association was found between LINC01291 and tumor stage (Fig. 1C), overall survival (Fig. 1D), and disease-free survival (Fig. 1E) in patients with melanoma.

To determine the role of LINC01291 in melanoma, its expression in melanoma cell lines (A-375, HT-144, SK-MEL-1, and A2058) was also measured via qRT-PCR. Compared with HEMs, all four melanoma cell lines exhibited higher LINC01291 expression (Fig. 1F), particularly in A-375 and HT-144 cell lines. Therefore, these two cell lines were subjected to transfection with si-LINC01291 to silence LINC01291 expression; the results were confirmed via qRT-PCR (Fig. 1G). To avoid off-target effects, si-LINC01291#1 and si-LINC01291#2 were used in functional studies. The CCK-8 and colony-forming assays revealed that cell proliferation (Fig. 1H) and colony formation (Fig. 1I) decreased in A-375 and HT-144 cells following LINC01291 knockdown. Furthermore, flow cytometry was used to determine the impact of LINC01291 silencing on melanoma cell apoptosis and cell cycle status. Notably, LINC01291 silencing promoted cell apoptosis (Fig. 1J) and cell cycle arrest at the G0/G1 stage (Fig. 1K) in A-375 and HT-144 cells. In addition, LINC01291 depletion triggered a decrease in A-375 and HT-144 cell migration (Fig. 1L) and invasion (Fig. 1M). The si-LINC01291#1 construct, which caused the most significant regulatory effects in melanoma cells, was selected for additional follow-up experiments. These findings suggest that LINC01291 functions as a pro-oncogenic lncRNA during melanoma etiology and progression.

### LINC01291 functions as a molecular sponge for miR-625-5p in melanoma cells

**Fig. 2:**
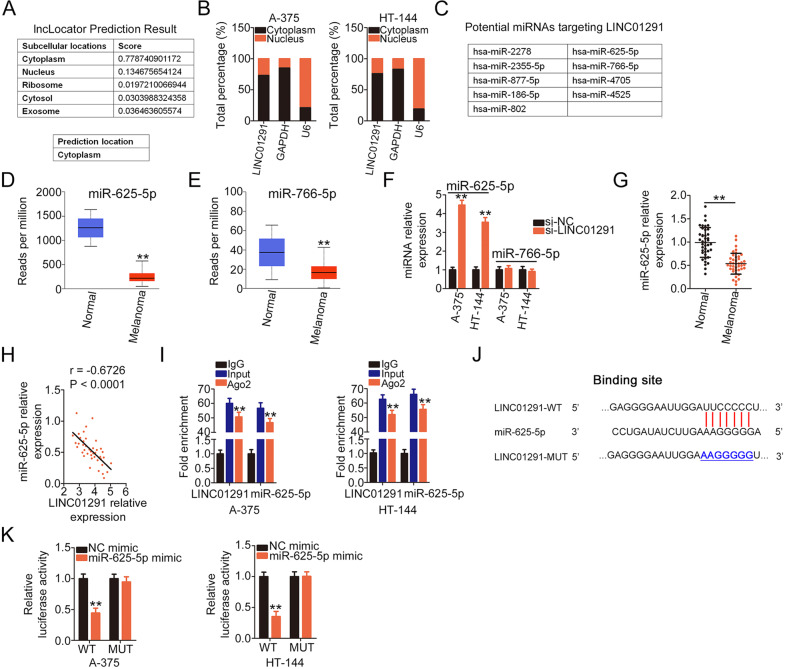
Long intergenic non-protein coding RNA 1291 (LINC01291) decreases miR-625-5p expression in melanoma cells by serving as a miR-625-5p sponge. **A** lncLocator predicted the localization of LINC01291. **B** Subcellular fractionation assay revealed that LINC01291 was mostly distributed in the cytoplasm of A-375 and HT-144 cells. **C** A total of nine miRNAs were identified via bioinformatics analysis to contain binding sequences for LINC01291. **D**, **E** Expression of miR-625-5p and miR-766-5p in melanoma, as analyzed using The Cancer Genome Atlas (TCGA) database. **P < 0.01 vs. group “Normal”. **F** Quantitative real-time polymerase chain reaction (qRT-PCR) was used to measure miR-625-5p and miR-766-5p expression in A-375 and HT-144 cells after LINC01291 interference. **P < 0.01 vs. group “si-NC”. **G** Total RNA was extracted from 41 pairs of human melanoma tissues and corresponding adjacent normal tissues and subjected to qRT-PCR for miR-625-5p quantification. **P < 0.01 vs. group “Normal”. **H** Pearson correlation analysis was used to analyze the association between miR-625-5p and LINC01291 expression in the 41 melanoma tissues. **I** Radioimmunoprecipitation (RIP) assay was performed to test the binding interaction between LINC01291 and miR-625-5p in melanoma cells. **P < 0.01 vs. group “IgG”. **J** The predicted binding sequences of miR-625-5p within LINC01291 and target-site mutation. **K** A-375 and HT-144 cells were transfected with miR-625-5p or negative control (NC) mimic along with LINC01291-WT or LINC01291-MUT. Luciferase activity was detected at 48 h after transfection. **P < 0.01 vs. group “NC mimic”.

Next, the regulatory mechanisms related to LINC01291 in melanoma cells were explored in further detail. lncLocator (http://www.csbio.sjtu.edu.cn/bioinf/lncLocator/) was used to predict the distribution of LINC01291. LINC01291 was found to be distributed in both the cytoplasm and the nucleus but was primarily located in the cytoplasm (Fig. 2A). The subcellular fractionation experiment further revealed the distribution of LINC01291 in the cytoplasm of A-375 and HT-144 cells (Fig. 2B). An increasing number of studies have described that lncRNAs may perform as ceRNAs or molecular sponges for certain miRNAs, such that these lncRNAs competitively bind to miRNAs and abolish their regulatory effects on target mRNAs [26–28]. Therefore, we speculated that LINC01291 executes its functions by serving as a miRNA sponge.

Using bioinformatics analysis, a total of nine miRNAs with targeted binding sites were identified (Fig. 2C). The expression of these candidate miRNAs in melanoma cells was examined using the TCGA database. miR-625-5p (Fig. 2D) and miR-766-5p (Fig. 2E) were downregulated in melanoma tissues, whereas the expression of the other miRNAs was either upregulated or unchanged (data not shown). qRT-PCR was then used to measure the expression of miR-625-5p and miR-766-5p in LINC01291-deficient A-375 and HT-144 cells. miR-625-5p was upregulated in A-375 and HT-144 cells after si-LINC01291 transfection (Fig. 2F). Additionally, miR-625-5p was weakly expressed in melanoma tissues (Fig. 2G) and exhibited an inverse correlation with LINC01291 expression (*r* = −0.6726, *P* < 0.0001; Fig. 2H). Furthermore, the RIP assay revealed that LINC01291 and miR-625-5p levels were both significantly enriched when incubated with Ago2 antibody than with control IgG (Fig. 2I). Figure 2J shows the WT and MUT binding sites of miR-625-5p within LINC01291. The luciferase reporter assay was used to confirm the binding between LINC01291 and miR-625-5p in melanoma cells. The luciferase activity of the LINC01291-WT reporter was significantly inhibited after miR-625-5p overexpression (Fig. 2K), whereas that of the LINC01291-MUT reporter remained unaltered. Collectively, the results suggest that LINC01291 functions as a miR-625-5p sponge in melanoma cells.

### miR-625-5p overexpression suppresses the malignant properties of melanoma cells

**Fig. 3:**
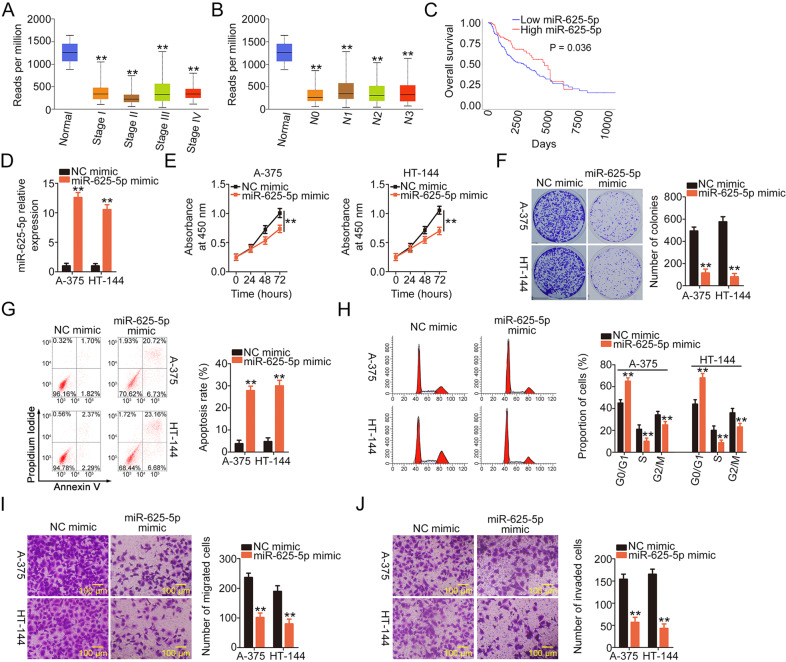
miR-625-5p overexpression suppresses melanoma cell proliferation, colony formation, migration, and invasion but promotes cell apoptosis and G0/G1 cell cycle arrest. **A**–**C** The Cancer Genome Atlas (TCGA) database was used to assess the association between miR-625-5p expression and tumor stage, lymph node metastasis, and overall survival in patients with melanoma. **P < 0.01 vs. group “Normal”. **D** miR-625-5p mimic or negative control (NC) mimic was introduced into A-375 and HT-144 cells. Quantitative real-time polymerase chain reaction (qRT-PCR) confirmed the transfection efficiency. **P < 0.01 vs. group “NC mimic”. **E**, **F** Cell proliferation and colony formation in A-375 and HT-144 cells after miR-625-5p overexpression was examined using Cell Counting Kit-8 (CCK-8) and colony formation assays. **P < 0.01 vs. group “NC mimic”. **G**, **H** Flow cytometry was performed to evaluate cell apoptosis and cell cycle status of miR-625-5p-overexpressing A-375 and HT-144 cells. **P < 0.01 vs. group “NC mimic”. **I**, **J** Cell migration and invasion of miR-625-5p mimic-transfected or NC mimic-transfected A-375 and HT-144 cells were investigated using cell migration and invasion assays. **P < 0.01 vs. group “NC mimic”.

As miR-625-5p is downregulated in melanoma, we sought to clarify the clinical value, biological role, and underlying mechanism of miR-625-5p in melanoma. The TCGA database revealed that low miR-625-5p expression correlated with tumor stage (Fig. 3A), and lymph node metastasis (Fig. 3B) in patients with melanoma. Additionally, patients with melanoma exhibiting low miR-625-5p expression had worse overall survival than those exhibiting with high miR-625-5p expression (*P* = 0.036; Fig. 3C).

To explore the role of miR-625-5p in melanoma, A-375 and HT-144 cells were transfected with a miR-625-5p mimic or NC mimic. qRT-PCR revealed that miR-625-5p expression in miR-625-5p mimic-transfected A-375 and HT-144 cells was significantly increased compared with than in NC mimic-transfected cells (Fig. 3D); this implies that the transfection was successful. The CCK-8 and colony-forming assays verified the inhibition of proliferation (Fig. 3E) and colony formation (Fig. 3F) in A-375 and HT-144 cells by miR-625-5p overexpression. Flow cytometry indicated that ectopic miR-625-5p expression accelerated the rate of apoptosis (Fig. 3G) and induced G0/G1 cell cycle arrest (Fig. 3H). Furthermore, the migration (Fig. 3I) and invasive (Fig. 3J) properties of A-375 and HT-144 cells after miR-625-5p mimic transfection was significantly impaired. Taken together, the results indicate that miR-625-5p plays a tumor-inhibiting role during melanoma progression.

### IGF-1R, a direct target of miR-625-5p, is regulated by LINC01291 by sponging miR-625-5p

**Fig. 4:**
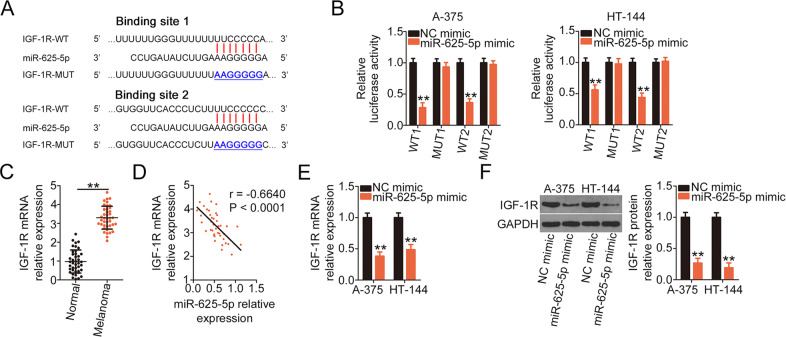
Insulin-like growth factor 1 receptor (IGF-1R) is a direct target of miR-625-5p in melanoma cells. **A** The potential binding sites between the 3′-untranslated regions (UTRs) and the mutant sequences of miR-625-5p and IGF-1R are shown. **B** Luciferase activity was determined in A-375 and HT-144 cells cotransfected with miR-625-5p mimic or negative control (NC) mimic and IGF-1R-WT or IGF-1R-MUT. **P < 0.01 vs. group “NC mimic”. **C** IGF-1R mRNA level was evaluated by quantitative real-time polymerase chain reaction (qRT-PCR) in 41 pairs of human melanoma tissues and corresponding adjacent normal tissues. **P < 0.01 vs. group “Normal”. **D** Correlation between miR-625-5p and IGF-1R expression in the 41 melanoma tissues was determined via Pearson correlation analysis. **E**, **F** mRNA and protein expression levels of IGF-1R in miR-625-5p-overexpressed A-375 and HT-144 cells was measured by qRT-PCR and western blotting, respectively. **P < 0.01 vs. group “NC mimic”.

To identify the mechanisms underlying the effects of miR-625-5p in melanoma, the putative target of miR-625-5p was identified by performing bioinformatics analysis. The binding sites of miR-625-5p within the 3′-UTR of IGF-1R and its mutant are presented in Fig. 4A. The luciferase activity of IGF-1R-WT (both 1 and 2) in A-375 and HT-144 cells was significantly suppressed after miR-625-5p mimic cotransfection (Fig. 4B), whereas no change in activity was observed in the IGF-1R-MUT group (both 1 and 2). Additionally, IGF-1R expression in melanoma tissues was measured via qRT-PCR, which confirmed that IGF-1R was highly expressed (Fig. 4C) and inversely associated with LINC01291 expression in melanoma tissues (*r* = −0.6640, *P* < 0.0001; Fig. 4D). Then, the regulatory effects of miR-625-5p on IGF-1R expression in melanoma cells were determined via qRT-PCR and western blotting. The data revealed that miR-625-5p upregulation decreased the mRNA (Fig. 4E) and protein (Fig. 4F) levels of IGF-1R in A-375 and HT-144 cells.

**Fig. 5:**
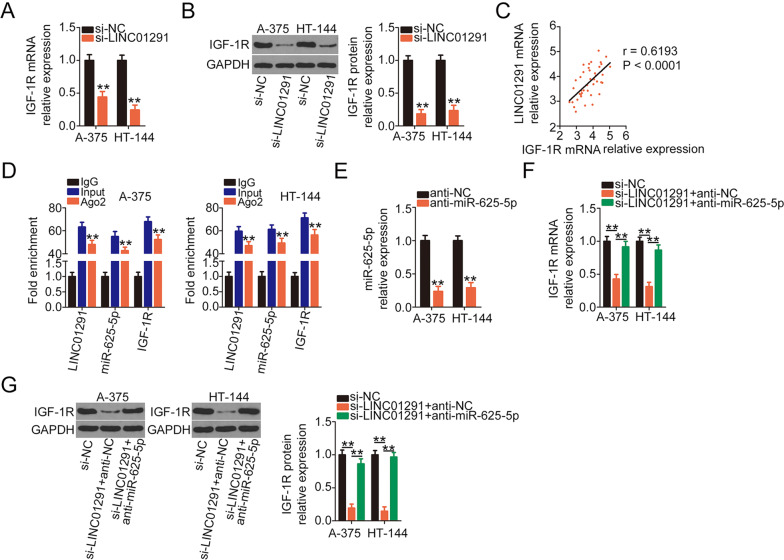
Long intergenic non-protein coding RNA 1291 (LINC01291) decoys miR-625-5p and promotes insulin-like growth factor 1 receptor (IGF-1R) expression in melanoma cells. **A**, **B** Quantitative real-time polymerase chain reaction (qRT-PCR) and western blotting were used to measure IGF-1R expression in A-375 and HT-144 cells after si-LINC01291 or non-targeted siRNA introduction. **P < 0.01 vs. group “si-NC”. **C** Pearson correlation analysis was performed to assess the association between LINC01291 and IGF-1R levels in the 41 melanoma tissues. **D** Radioimmunoprecipitation (RIP) assay was conducted to validate the binding interaction between LINC01291, miR-625-5p, and IGF-1R in melanoma cells. **P < 0.01 vs. group “IgG”. **E** qRT-PCR was used to explore the efficiency of anti-miR-625-5p transfection in A-375 and HT-144 cells. **P < 0.01 vs. group “anti-NC”. **F**, **G** Anti-miR-625-5p or anti-NC was transfected into LINC01291-deficient A-375 and HT-144 cells. The changes in the mRNA and protein expression levels of IGF-1R were determined via qRT-PCR and western blotting, respectively. **P < 0.01 vs. groups “si-NC” and “si-LINC01291 + anti-NC”.

To confirm the interaction among LINC01291, miR-625-5p, and IGF-1R in melanoma, the expression of IGF-1R in LINC01291-deficient A-375 and HT-144 cells was determined. qRT-PCR and western blotting revealed that si-LINC01291 transfection resulted in a decrease in IGF-1R expression in A-375 and HT-144 cells at the mRNA (Fig. 5A) and protein (Fig. 5B) levels. Correlation analysis further revealed a positive correlation between LINC01291 and IGF-1R expression in melanoma tissues (*r* = 0.6193, *P* < 0.0001; Fig. 5C). Notably, the enrichment of LINC01291, miR-625-5p, and IGF-1R was enhanced in Ago2-immunoprecipitated RNA than in IgG-immunoprecipitated RNA (Fig. 5D), suggesting that these three molecules are present in the same RNA-induced silencing complex. A series of experiments were designed to evaluate whether sequestering miR-625-5p is necessary for the regulatory activities of LINC01291 on IGF-1R in melanoma cells. Anti-miR-625-5p or anti-NC along with si-LINC01291 was cotransfected into A-375 and HT-144 cells and IGF-1R expression was measured. The efficiency of anti-miR-625-5p in reducing miR-625-5p expression was confirmed via qRT-PCR (Fig. 5E). Interestingly, the mRNA (Fig. 5F) and protein (Fig. 5G) expression levels of IGF-1R were significantly downregulated in LINC01291-deficient-A-375 and HT-144 cells; however, IGF-1R expression recovered after anti-miR-625-5p cotransfection. Therefore, it is safe to conclude that LINC01291 sponges miR-625-5p and subsequently upregulates IGF-1R expression in melanoma cells.

### Increased activity of the miR-625-5p/IGF-1R axis counteracts the inhibitory effects of LINC01291 silencing in melanoma cells

**Fig. 6:**
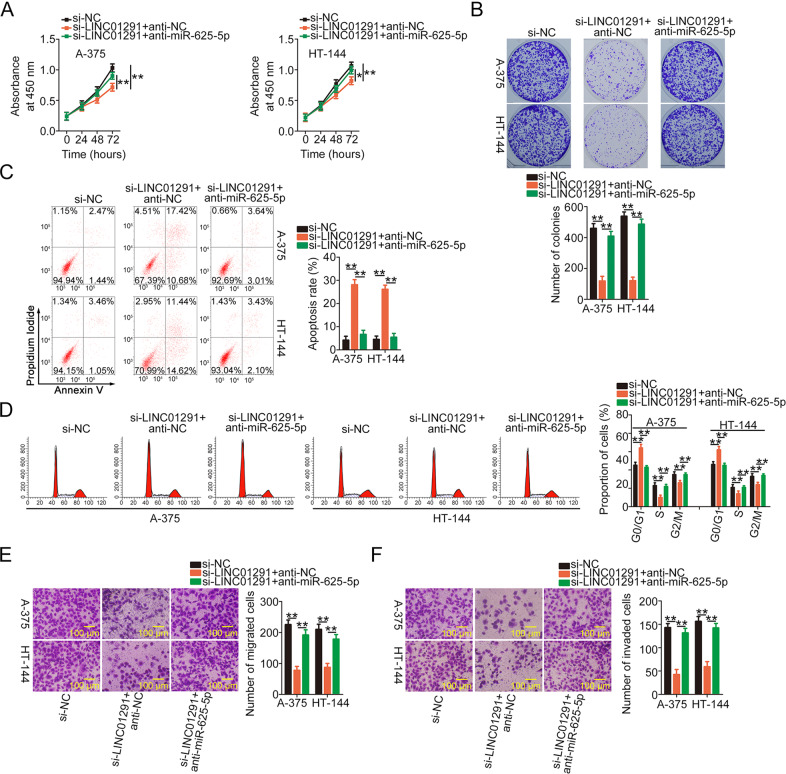
miR-625-5p inhibition rescues the antioncogenic effects of long intergenic non-protein coding RNA 1291 (LINC01291) knockdown in melanoma cells. **A**, **B** Cell Counting Kit-8 (CCK-8) and colony-forming assays reflected the proliferation and colony formation ability of A-375 and HT-144 cells after cotransfection with anti-miR-625-5p or anti-NC and si-LINC01291. *P < 0.05 and **P < 0.01 vs. groups “si-NC” and “si-LINC01291 + anti-NC”. **C**, **D** Apoptosis and cell cycle status of A-375 and HT-144 cells were detected via flow cytometry following transfection with anti-miR-625-5p or anti-NC in combination with si-LINC01291. **P < 0.01 vs. groups “si-NC” and “si-LINC01291 + anti-NC”. **E**, **F** Cell migration and invasion assays were performed to determine the migration and invasive capabilities of A-375 and HT-144 cells treated as described above. **P < 0.01 vs. groups “si-NC” and “si-LINC01291 + anti-NC”.

Functional rescue experiments were conducted to determine whether LINC01291 affects tumor processes in melanoma cells by decoying miR-625-5p and increasing IGF-1R. LINC01291-depleted A-375 and HT-144 cells were cotransfected with anti-miR-625-5p or anti-NC. LINC01291 interference impaired the proliferation (Fig. 6A) and colony-forming ability (Fig. 6B) of A-375 and HT-144 cells; these effects were partially abolished upon miR-625-5p inhibition. Additionally, miR-625-5p downregulation counteracted the LINC01291 silencing-mediated increase in apoptosis (Fig. 6C) and induced G0/G1 cell cycle arrest (Fig. 6D). Furthermore, the migration (Fig. 6E) and invasion (Fig. 6F) of A-375 and HT-144 cells were inhibited by LINC01291 loss; however, the effects were reversed after anti-miR-625-5p cotransfection.

**Fig. 7:**
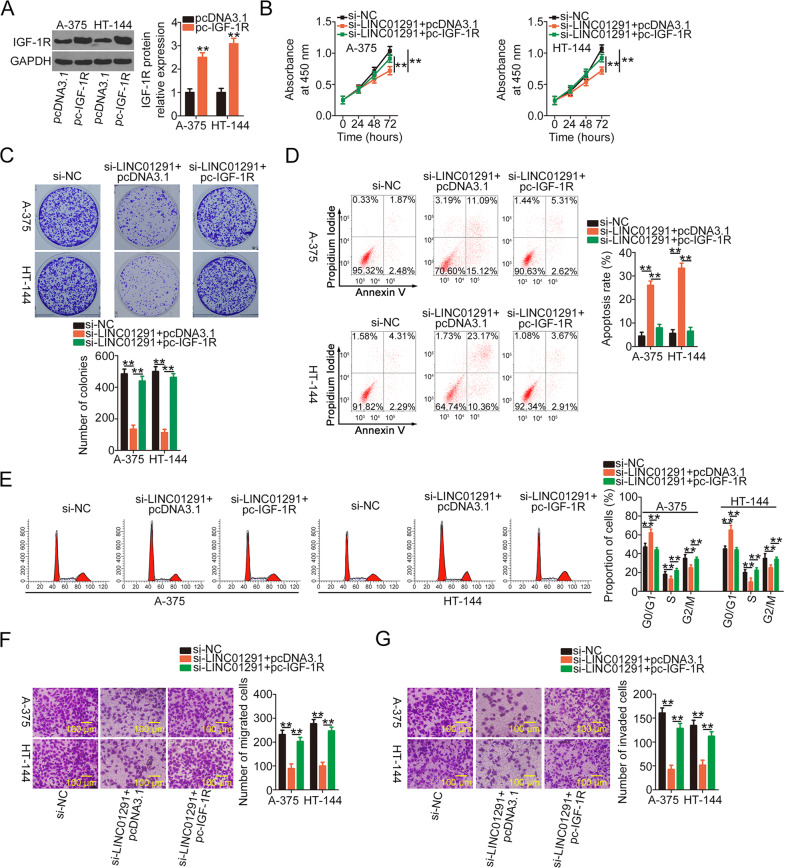
Overexpressing insulin-like growth factor 1 receptor (IGF-1R) counteracts the effects of long intergenic non-protein coding RNA 1291 (LINC01291) silencing in melanoma cells. **A** Western blotting was performed to detect the expression of IGF-1R protein in A-375 and HT-144 cells after pc-IGF-1R or pcDNA3.1 transfection. **P < 0.01 vs. “pcDNA3.1” group. **B**, **C** Cell Counting Kit-8 (CCK-8) and colony formation assays were conducted to examine the proliferation and colony formation ability of A-375 and HT-144 cells after cotransfection of pc-IGF-1R or pcDNA3.1 and si-LINC01291. **P < 0.01 vs. groups “si-NC” and “si-LINC01291 + pcDNA3.1”. **D**, **E** Flow cytometry was performed to measure the apoptotic rate and cell cycle status of the aforementioned cells. **P < 0.01 vs. groups “si-NC” and “si-LINC01291 + pcDNA3.1”. **F**, **G** Cell migration and invasive abilities of cells treated with si-LINC01291 along with pc-IGF-1R or pcDNA3.1 were observed using cell migration and invasion assays. **P < 0.01 vs. groups “si-NC” and “si-LINC01291 + pcDNA3.1”.

Next, the IGF-1R expression plasmid, pc-IGF-1R, was introduced into A-375 and HT-144 cells that were previously transfected with si-LINC01291. Western blotting verified the transfection efficiency of pc-IGF-1R (Fig. 7A). As expected, the re-introduction of IGF-1R abrogated the regulatory effects of si-LIN01291 on A-375 and HT-144 cell proliferation (Fig. 7B), colony formation (Fig. 7C), apoptosis (Fig. 7D), and cell cycle status (Fig. 7E). Consistent with these findings, cell migration and invasion assays revealed decreased migration (Fig. 7F) and invasion (Fig. 7G) of si-LIN01291-transfected A-375 and HT-144 cells, which was again neutralized in response to pc-IGF-1R cotransfection. In general, these results suggest that LINC01291 exhibits pro-oncogenic roles in melanoma cells by targeting the miR-625-5p/IGF-1R axis.

### LINC01291 downregulation restricts the growth of melanoma tumors in vivo

**Fig. 8:**
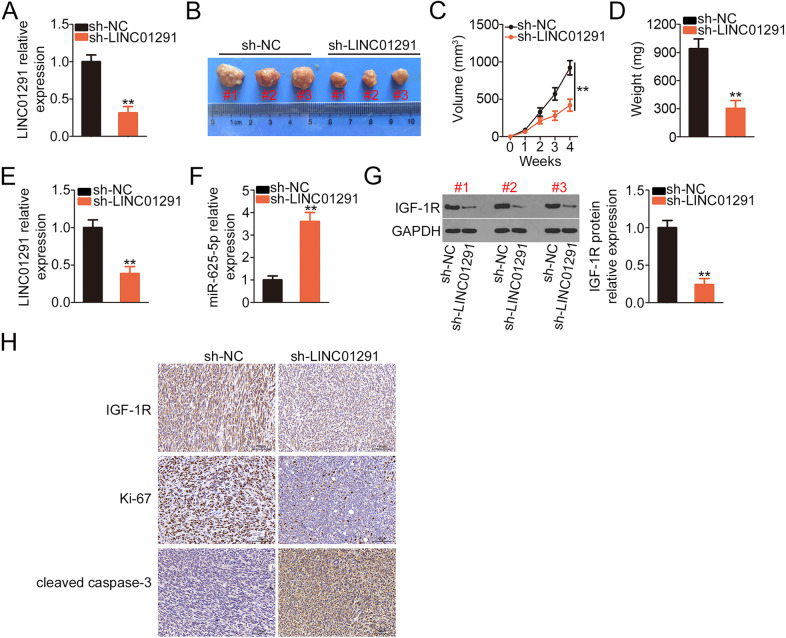
Loss of long intergenic non-protein coding RNA 1291 (LINC01291) inhibits melanoma tumorigenesis in vivo. **A** Quantitative real-time polymerase chain reaction (qRT-PCR) was performed to measure LINC01291 expression in A-375 cells after infection with sh-LINC01291 or sh-NC. **P < 0.01 vs. “sh-NC” group. **B** Image of the tumor xenografts obtained from the sh-LINC01291 and sh-NC groups. **C** Tumor volumes were monitored every week and the tumor volume–time curves were plotted. **P < 0.01 vs. “sh-NC” group. **D** At 4 weeks after injection, tumor weight was measured. **P < 0.01 vs. “sh-NC” group. **E**–**G** LINC01291, miR-625-5p, and IGF-1R levels in tumor xenografts were detected. **P < 0.01 vs. “sh-NC” group. **H** Immunohistochemistry was performed to measure the expression of IGF-1R, Ki-67, and cleaved caspase-3 in the tumor xenografts.

To examine the impact of LINC01291 on tumor growth in vivo, A-375 cells expressing stable sh-LINC01291 or sh-NC were subcutaneously injected into nude mice. The efficiency of sh-LINC01291 transfection was determined via qRT-PCR. The results showed that sh-LINC01291 was capable of decreasing LINC01291 expression in A-375 cells (Fig. 8A). Tumor volume (Fig. 8B, C) and weight (Fig. 8D) decreased in the sh-LINC01291 group compared with those in the sh-NC group. Subsequently, the expression levels of LINC01291, miR-625-5p, and IGF-1R were measured in tumor xenografts. The data indicate that LINC01291 (Fig. 8E) was downregulated, whereas miR-625-5p (Fig. 8F) was upregulated in the tumor xenografts originating from sh-LINC01291-transfected A-375 cells compared with those originating from sh-NC-transfected cells. The protein level of IGF-1R (Fig. 8G) decreased in the tumor xenografts with LINC01291 depletion. Furthermore, tumor xenografts with LINC01291 depletion presented decreased Ki-67 and IGF-1R expression as well as increased cleaved caspase-3 expression (Fig. 8H). Taken together, our data demonstrate the suppressive effects of LINC01291 on the in vivo tumorigenesis of melanoma.

### The absence of LINC01291 lowers the chemoresistance of melanoma cells to cisplatin

**Fig. 9:**
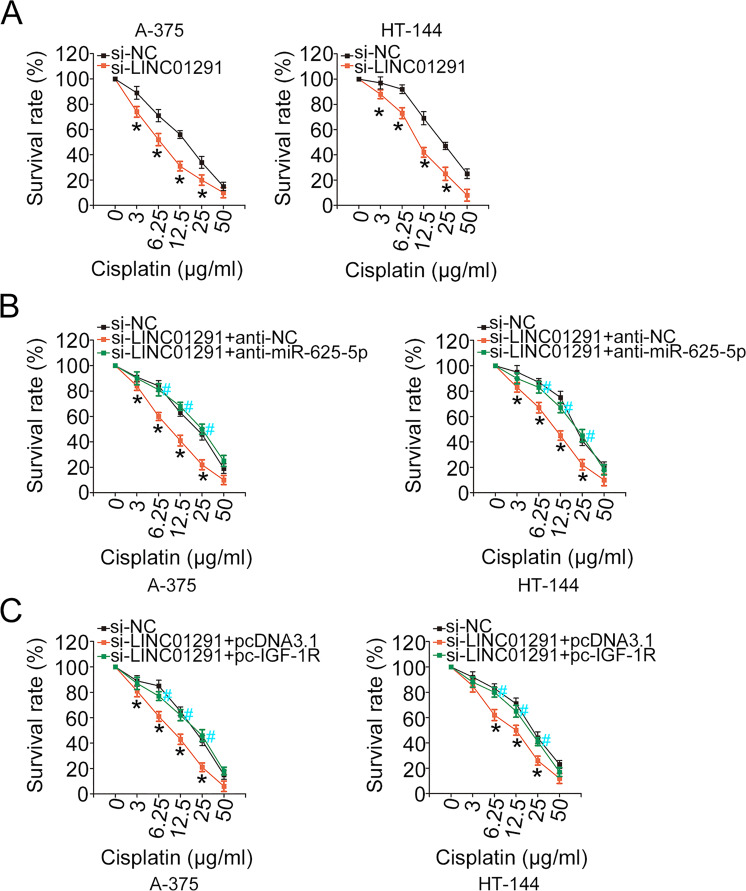
Knockdown of long intergenic non-protein coding RNA 1291 (LINC01291) inhibits the chemoresistance of melanoma cells to cisplatin. **A** A-375 and HT-144 cells transfected with either si-LINC01291 or si-NC were incubated with cisplatin for 48 h. CCK-8 assay was performed to determine whether LINC01291 can affect the chemoresistance of A-375 and HT-144 cells to cisplatin. **P < 0.01 vs. group si-NC. **B** The LINC01291-silenced A-375 and HT-144 cells were transfected with anti-NC or anti-miR-625-5p. After incubation with cisplatin for 48 h, the CCK-8 assay was performed to determine the survival rate in different groups. **P < 0.01 vs. group “si-NC”. ^#^*P* < 0.01 vs. group “si-LINC01291 + anti-miR-625-5p”. **C** A-375 and HT-144 cells were cotransfected with pc-IGF-1R or pcDNA3.1 and si-LINC01291. After incubation with cisplatin for 48 h, the CCK-8 assay was performed to determine the survival rate in different groups. **P < 0.01 vs. group “si-NC”. ^#^*P* < 0.01 vs. group “si-LINC01291 + pcDNA3.1”.

To address the roles of LINC01291 on chemoresistance of melanoma cells to cisplatin, LINC01291-silenced A-375 and HT-144 cells were further cultivated with different concentrations of cisplatin for 48 consecutive hours. A-375 and HT-144 cells transfected with si-LINC01291 presented an obviously decreased survival rate after they were treated with cisplatin (Fig. 9A), suggesting that loss of LINC01291 decreased the chemoresistance of melanoma cells to cisplatin. Further, rescue experiments were implemented to assess whether miR-625-5p/IGF-1R axis contributes to the effect of LINC01291 on chemoresistance of melanoma cells to cisplatin. Inhibiting miR-625-5p (Fig. 9B) or upregulating IGF-1R (Fig. 9C) could offset the regulatory effects of si-LINC01291 on A-375 and HT-144 cell chemosensitivity to cisplatin. Thus, LINC01291 silencing weakens the resistance of melanoma cells to cisplatin by regulating the miR-625-5p/IGF-1R axis.

## Discussion

A number of lncRNAs have been shown to be differentially expressed in melanoma [29–31]. Several studies have also validated the relationship between dysregulated lncRNAs and melanoma pathogenesis, with these regulatory actions being mediated by multiple mechanistic interactions [32–34]. Therefore, studying melanoma-related lncRNAs and identifying their specific functions are useful strategies to identify putative targets for melanoma therapy. However, there are still many lncRNAs whose expressions and detailed roles remain largely unclear in melanoma. In the present study, we measured LINC01291 expression in melanoma and further explored the role of LINC01291 in regulating tumor behavior. Finally, the molecular events via which LINC01291 exerts its effects on melanoma cells were investigated.

Previous studies have reported that lncRNAs contribute to melanoma etiology and progression. For instance, SNHG16 [35], TTN-AS1 [36], and MALAT1 [37] are overexpressed in melanoma and exert oncogenic effects. In contrast, FENDRR [18], PINT [38], and LINC00459 [39] are weakly expressed in melanoma and execute antioncogenic roles by inhibiting malignant processes. However, to the best of our knowledge, the expression profile and functions of LINC01291 in melanoma remain unclear. In this study, a significant upregulation in LINC01291 expression was observed in both melanoma tissues and cell lines. By silencing LINC01291 in melanoma cells, cell proliferation, colony formation, migration, and invasion were inhibited, whereas apoptosis was enhanced and the cell cycle was arrested at the G0/G1 stage. Additionally, LINC01291 knockdown decreased the chemoresistance of melanoma cells to cisplatin in vitro and inhibited the growth of melanoma tumors in vivo. Therefore, our study identified LINC01291 as a tumor-promoting lncRNA in melanoma for the first time.

lncRNAs are involved in carcinogenesis and cancer progression via a variety of different mechanisms [40]. At the transcriptional level, lncRNAs can epigenetically knockdown mRNAs [41]. At the post-transcriptional level, the ceRNA network has received widespread concern in the study of lncRNAs [22]. The ceRNA theory proposes that lncRNA have the power to serve as ceRNA of certain miRNA and impede the interaction between miRNAs and target mRNAs, subsequently impairing the miRNA targeting activity on mRNA [22, 42, 43]. Therefore, we verified LINC01291 localization using lncLocator and a subcellular fractionation assay. The data identified LINC01291 as a cytoplasmic lncRNA in melanoma cells. Then, we attempted to address whether LINC01291 could function as a miRNA sponge in melanoma.

Using bioinformatics analysis, LINC01291 was found to contain a binding site for miR-625-5p. Molecular analysis indicated that LINC01291 loss increased miR-625-5p expression in melanoma cells. In addition, correlation analysis revealed a negative correlation between LINC01291 and miR-625-5p levels in melanoma tissues. More importantly, LINC01291 directly binds to and interacted with miR-625-5p, as determined via luciferase reporter and RIP assays. IGF-1R was verified as the direct target of miR-625-5p and was positively modulated by LINC01291 in melanoma cells via miR-625-5p sponging. All these results identified a new ceRNA pathway in melanoma, which consists of LINC01291, miR-625-5p, and IGF-1R.

miR-625-5p is downregulated in melanoma and exerts an inhibitory effect on oncogenicity [44, 45]. In this study, we found that decreased miR-625-5p levels were associated with tumor stage and lymph node metastasis in melanoma. Additionally, Kaplan–Meier survival analysis revealed that patients with melanoma characterized with low miR-625-5p expression exhibit poor prognosis. Moreover, mechanical experiments supported IGF-1R as a direct target of miR-625-5p in melanoma cells. IGF-1R was further shown to be negatively regulated by miR-625-5p and positively regulated by LINC01291. Rescue experiments revealed that the functions caused by LINC01291 downregulation in melanoma cells could be reversed by suppressing miR-625-5p or overexpressing IGF-1R. Therefore, our results corroborate that LINC01291 exhibits its tumor-promoting effects during melanoma progression by targeting the miR-625-5p/IGF-1R axis.

Our study had two limitations. First, we did not examine the expression profile of LINC01291, and its biological function in basal cell carcinoma. In the near future, we will collect basal cell carcinoma and matched non-cancerous skin, and then determined whether LINC01291 is implicated in the oncogenesis and progression of this skin cancer. Second, our study only uncovered that LINC01291 silencing decreased the chemoresistance of melanoma cells to cisplatin. The next step of our study will induce a drug-resistant melanoma cell line in our laboratory. Next, the expression profile and biological impacts of LINC01291 on drug-resistant melanoma cells will be unveiled.

In summary, our study confirmed the involvement of LINC01291, miR-625-5p, and IGF-1R in melanoma and identified the LINC01291/miR-625-5p/IGF-1R ceRNA pathway. Further, our results demonstrated that LINC01291 promotes the oncogenicity of melanoma cells by serving as a molecular sponge for miR-625-5p, thereby reinforcing IGF-1R expression. The LINC01291/miR-625-5p/IGF-1R signaling pathway may represent a novel therapeutic target for melanoma therapy.
